# Roles of epidermal growth factor receptor, claudin-1 and occludin in multi-step entry of hepatitis C virus into polarized hepatoma spheroids

**DOI:** 10.1371/journal.ppat.1011887

**Published:** 2023-12-29

**Authors:** Chui-Wa So, Marion Sourisseau, Shamila Sarwar, Matthew J. Evans, Glenn Randall

**Affiliations:** 1 Department of Microbiology, The University of Chicago, Chicago, Illinois, United States of America; 2 Department of Microbiology, Icahn School of Medicine at Mount Sinai, New York, New York, United States of America; Princeton University, UNITED STATES

## Abstract

The multi-step process of hepatitis C virus (HCV) entry is facilitated by various host factors, including epidermal growth factor receptor (EGFR) and the tight junction proteins claudin-1 (CLDN1) and occludin (OCLN), which are thought to function at later stages of the HCV entry process. Using single particle imaging of HCV infection of polarized hepatoma spheroids, we observed that EGFR performs multiple functions in HCV entry, both phosphorylation-dependent and -independent. We previously observed, and in this study confirmed, that EGFR is not required for HCV migration to the tight junction. EGFR is required for the recruitment of clathrin to HCV in a phosphorylation-independent manner. EGFR phosphorylation is required for virion internalization at a stage following the recruitment of clathrin. HCV entry activates the RAF-MEK-ERK signaling pathway downstream of EGFR phosphorylation. This signaling pathway regulates the sorting and maturation of internalized HCV into APPL1- and EEA1-associated early endosomes, which form the site of virion uncoating. The tight junction proteins, CLDN1 and OCLN, function at two distinct stages of HCV entry. Despite its appreciated function as a “late receptor” in HCV entry, CLDN1 is required for efficient HCV virion accumulation at the tight junction. Huh-7.5 cells lacking CLDN1 accumulate HCV virions primarily at the initial basolateral surface. OCLN is required for the late stages of virion internalization. This study produced further insight into the unusually complex HCV endocytic process.

## Author summary

Hepatitis C virus (HCV) is a hepatotropic RNA virus. An estimated 58 million people are chronically infected with HCV, while HCV-associated hepatocellular carcinoma and cirrhosis accounted for 290, 000 deaths in 2019. HCV entry into hepatocytes is highly complex with distinct stages and multiple host cofactors. Perturbing the interaction of HCV with entry host factors is potential strategy for therapeutic and vaccine-based anti-HCV approaches. However, the precise functions of most of the host factors in HCV entry are not fully appreciated. Moreover, studies of entry using polarized cell culture that physiologically resembles hepatocytes in vivo are limited. In this paper, we evaluate the roles of three entry factors in polarized hepatoma spheroids: epidermal growth factor receptor (EGFR) and tight junction proteins claudin-1 (CLDN1) and occludin (OCLN). We observe that EGFR performs multiple functions to regulate HCV internalization and trafficking to endosomes. The process involves EGFR phosphorylation-dependent and -independent roles and downstream RAF-MEK-ERK signaling pathway. We also show that CLDN1 and OCLN, despite both localizing at the tight junction, function at two distinct stages of HCV entry. CLDN1 is required for the accumulation of HCV virions at the tight junction from the basolateral membrane, while OCLN regulates HCV internalization. These findings provide insights into the mechanism of HCV entry and highlight potential interventional strategies.

## Introduction

Hepatitis C virus (HCV) is an enveloped, positive-sense RNA virus of the *Flaviviridae* family. It has a specific tissue tropism, infecting hepatocytes preferentially. Hepatocytes are polarized with two distinct membrane domains. The apical domains of adjacent hepatocytes form the bile canaliculus into which bile is secreted, while the basolateral domain faces the sinusoidal endothelium and regulates the exchange of materials with the circulating blood. Tight junction proteins separate the two domains and perform a barrier function excluding bile from the circulating blood [1–4].

HCV entry is a complex and multi-step process. The HCV virion contains host-derived lipids and apolipoproteins (Apos) that are acquired during assembly [5–7]. Interactions of the lipids and Apos with attachment factors, such as low-density lipoprotein receptor [8] and glycosaminoglycans [9], facilitate initial binding of the virion to hepatocytes. Cluster of differentiation 81 (CD81), scavenger receptor BI (SR-BI), and tight junction proteins claudin-1 (CLDN1) and occludin (OCLN) are crucial host cofactors of HCV entry. Expressing all four proteins together renders non-permissive HEK293T cells susceptible to pseudotyped HCV particles (HCVpp). CD81 and OCLN also contribute to the species specificity of HCV infection [10].

HCV envelope glycoprotein E2 binds to CD81 [11] and SR-BI [12]. Studies suggest that CD81 acts prior to CLDN1, while CLDN1 acts prior to OCLN during HCV entry [13–15]. While studies have shown that CLDN1 and OCLN are co-immunoprecipitated with HCV E2, so far there is no evidence of direct binding on the cell surface [16,17]. CLDN1 mutants that are expressed on the plasma membrane but not exclusively at the cell-cell contact are less efficient in rendering non-permissive cells infectable with HCVpp [17]. This suggests that the tight junctional localization of CLDN1 is crucial for its function during HCV entry. The first extracellular loop of CLND1 regulates its interaction with CD81 and HCV entry [13,18–20]. For OCLN, the second extracellular loop is required for HCV entry and contributes to the species tropism of HCV [10,21,22].

Epidermal growth factor receptor (EGFR), a receptor tyrosine kinase, is an additional entry cofactor [23]. EGFR is phosphorylated during HCV entry and phosphorylation of tyrosine residues 1147 and 1173 are required for HCV infection [24]. The RAF-MEK-ERK signaling pathway downstream of EGFR is activated during HCV infection [25,26]. However, whether the activation occurs during HCV entry into polarized spheroids has not been tested. Inhibitors targeting the pathway reduced HCV entry at a post-binding step [27,28]. However, the specific function of the RAF-MEK-ERK pathway in HCV endocytosis is unknown. The virion is internalized via clathrin-mediated endocytosis [29,30] and sorted into Rab5-positive early endosomes to undergo fusion [24,31].

We previously developed single particle tracking of HCV in polarized hepatoma spheroids. HCV virions were labeled with the lipophilic fluorescent dye DiD and purified by density gradient ultracentrifugation to enrich for highly infectious virions. DiD particles colocalized with HCV core, E2, and ApoE, demonstrating specific labeling of virions [24,32]. We showed by electron microscopy that DiD-HCV particles were pure virions not contained within exosomes [24]. Matrigel-embedded Huh-7.5 spheroids displayed polarized localization of apical, basolateral, and tight junctional markers. The spheroids retained the bile analog 5-chloromethyfluorescein diacetate (CMFDA) at the apical domains, suggesting that the spheroids showed functional characteristics of the liver [24].

Using single particle imaging of HCV in spheroids, we previously described sequential events in HCV entry. First, HCV virions migrate from the basolateral membrane to the tight junction in association with the early entry factors CD81, SR-BI, and EGFR in an actin-dependent manner. Then, HCV virions are internalized via clathrin-mediated endocytosis at the tight junction in an EGFR-dependent manner [24]. These two distinct steps of HCV entry were not previously observed in two-dimensional monolayer cell culture, due to its poor resemblance of hepatocyte polarity. Moreover, we showed that EGFR is not required for HCV migration to the tight junction, which is different from what has been inferred from studies in unpolarized hepatocytes [28]. Currently, studies of HCV infection using polarized cell culture systems are limited [33].

In this study, we characterized the functions of the late host entry factors: epidermal growth factor receptor (EGFR), claudin-1 (CLDN1), and occludin (OCLN) during HCV entry into polarized hepatoma spheroids. In order to distinguish between EGFR phosphorylation-dependent and -independent functions, we used two well-characterized pharmacological inhibitors that specifically target distinct stages of EGFR signaling: AG-1478 and sorafenib. AG-1478 is an EGFR kinase inhibitor commonly used in studies of the cellular processes and oncogenic activities of EGFR. The inhibitor competes with ATP to bind to lysine residue 721 of EGFR. It induces reversible EGFR dimerization with no associated kinase activity [34–37]. Sorafenib specifically targets RAF kinase in the RAF-MEK-ERK signaling pathway downstream of EGFR. The inhibitor occupies the ATP binding pocket and catalytic motifs of RAF [38]. It significantly inhibits the kinase activity of RAF but not that of EGFR, MEK, or ERK [39]. It has no effect on the activation of the PI3K-AKT pathway, which is another downstream signaling pathway mediated by EGFR [40].

We find that EGFR performs multiple functions during HCV internalization into polarized hepatoma spheroids. EGFR initially regulates the recruitment of clathrin to HCV virions. In this process, EGFR phosphorylation is dispensable. EGFR phosphorylation regulates virion internalization at a stage following clathrin recruitment. HCV entry also activates the RAF-MEK-ERK pathway downstream of EGFR phosphorylation. This signaling pathway is required for the recruitment of early endosomal proteins APPL1 and EEA1 to clathrin-coated vesicles containing internalized HCV. CLDN1 is required for the accumulation of HCV virions at the tight junction. OCLN is required for the stabilization of clathrin coated pits and subsequent HCV internalization.

## Results

### Distinct EGFR signaling pathways induced during HCV entry are inhibited by AG-1478 and sorafenib

EGFR phosphorylation regulates multiple downstream signaling pathways [41]. We previously showed that EGFR Y1148 and Y1173 were phosphorylated and required for HCV infection [24]. Phosphorylated Y1148 and Y1173 interact with adaptor proteins Grb2 and SHC [42,43]. Activated SHC provides an additional binding site for Grb2 [44]. Grb2 then activates the downstream signaling pathway RAF-MEK-ERK [41]. siRNA silencing of SHC inhibited HCV infection, suggesting that the RAF-MEK-ERK pathway is required for HCV infection [28].

**Fig 1:**
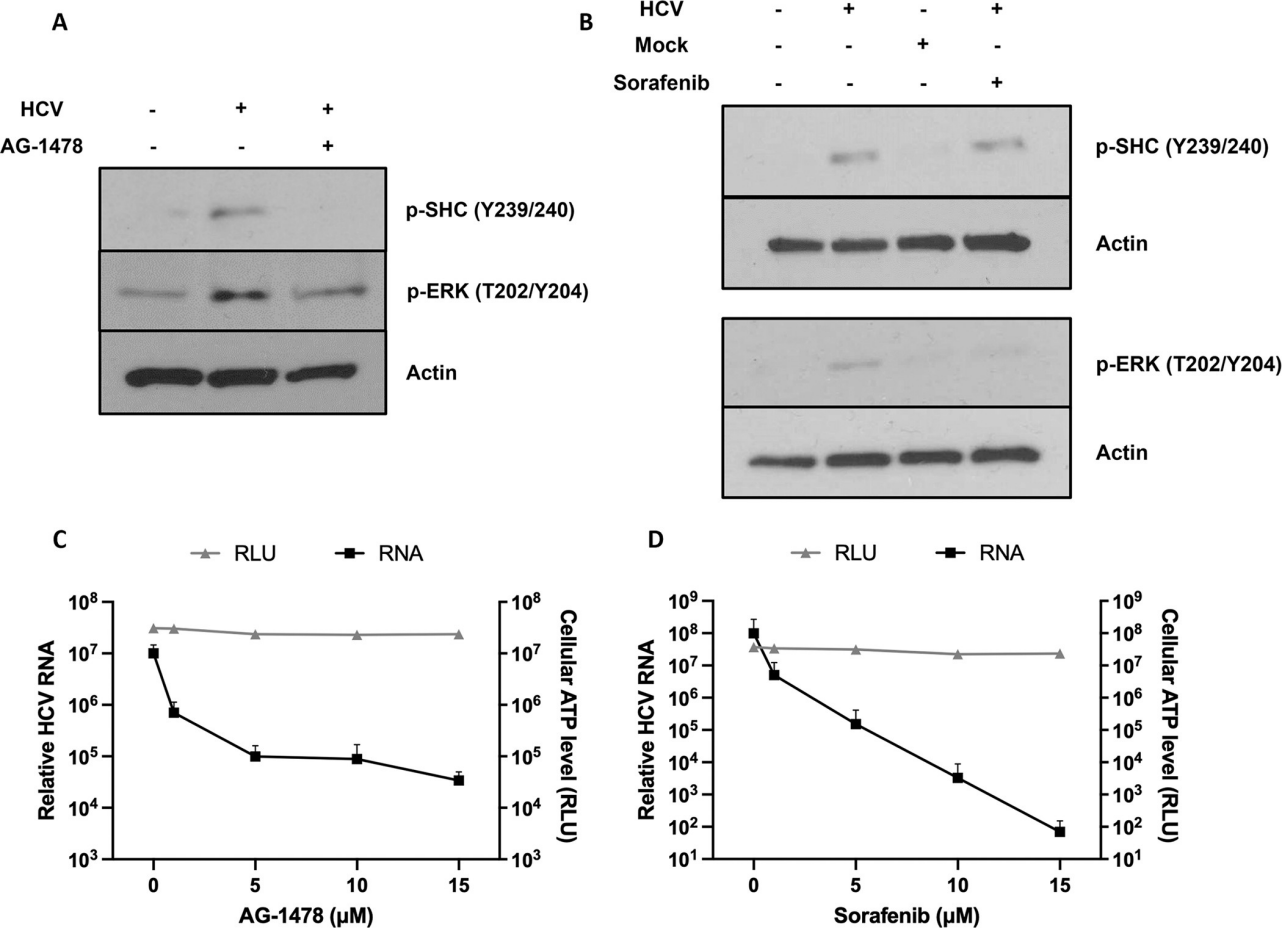
HCV infection activates the RAF-MEK-ERK signaling pathway via EGFR phosphorylation. (A and B) Huh-7.5 spheroids were serum starved, incubated with 5 μM AG-1478 (A) or sorafenib (B) for 2 hr if indicated, infected with concentrated HCV with 5 μM AG-1478 (A) or sorafenib (B) for 1 hr at 4°C, shifted to 37°C, processed with Matrigel cell recovery solution, and lysed at 120 min post temperature shift. Lysate samples were immunoblotted for the indicated proteins. (C and D) Huh-7.5 cells were seeded onto 96-well plates, incubated with AG-1478 (C) or sorafenib (D) for 2 hr, infected with HCV with AG-1478 (C) or sorafenib (D) for 22 hr, and then analyzed for relative HCV RNA levels. To examine cell viability, Huh-7.5 cells were seeded onto 96-well plates, incubated with AG-1478 (C) or sorafenib (D) for 24 hr, and then analyzed for cellular ATP levels. Mean +/- SD.

We first examined whether components of the RAF-MEK-ERK pathway were activated upon HCV entry into hepatoma spheroids. Huh-7.5 spheroids were infected with HCV at 4°C to synchronize infection, then incubated at 37°C for 120 min post temperature shift to examine the phosphorylation of SHC and ERK (Figs 1A, 1B and S1A). Phosphorylation of SHC at Y239/240 and ERK at T202/Y204 were increased in infected spheroids relative to uninfected or mock infected spheroids (Fig 1B). AG-1478 pre-treatment inhibited EGFR phosphorylation induced by EGF (S1B Fig) and inhibited HCV-stimulated phosphorylation of both ERK and SHC, suggesting that HCV entry activates SHC/ERK in an EGFR phosphorylation-dependent manner (Fig 1A).

To investigate whether the RAF-MEK-ERK pathway was required for HCV entry, we used the RAF inhibitor sorafenib. Sorafenib occupies the ATP binding pocket and catalytic motifs of RAF [38]. It significantly inhibits the kinase activity of RAF but not that of EGFR, MEK, or ERK [39]. Sorafenib inhibited HCV stimulated phosphorylation of ERK, but not SHC, demonstrating the selectivity of this inhibitor in analyzing the RAF/ERK pathway in HCV infection (Fig 1B). Both AG-1478 and sorafenib pre-treatment of the Huh-7.5 spheroids significantly inhibited RNA replication without impacting cell viability (Fig 1C and 1D) [6]. Importantly, when HCV endocytosis was bypassed via electroporation of HCV RNA, AG-1478 and sorafenib had no effect on infectious virus production, demonstrating that their antiviral effects were due to inhibition of HCV entry and not perturbing later stages of the viral life cycle (S1C Fig).

### EGFR phosphorylation regulates HCV internalization but is not required for the recruitment of clathrin

**Fig 2:**
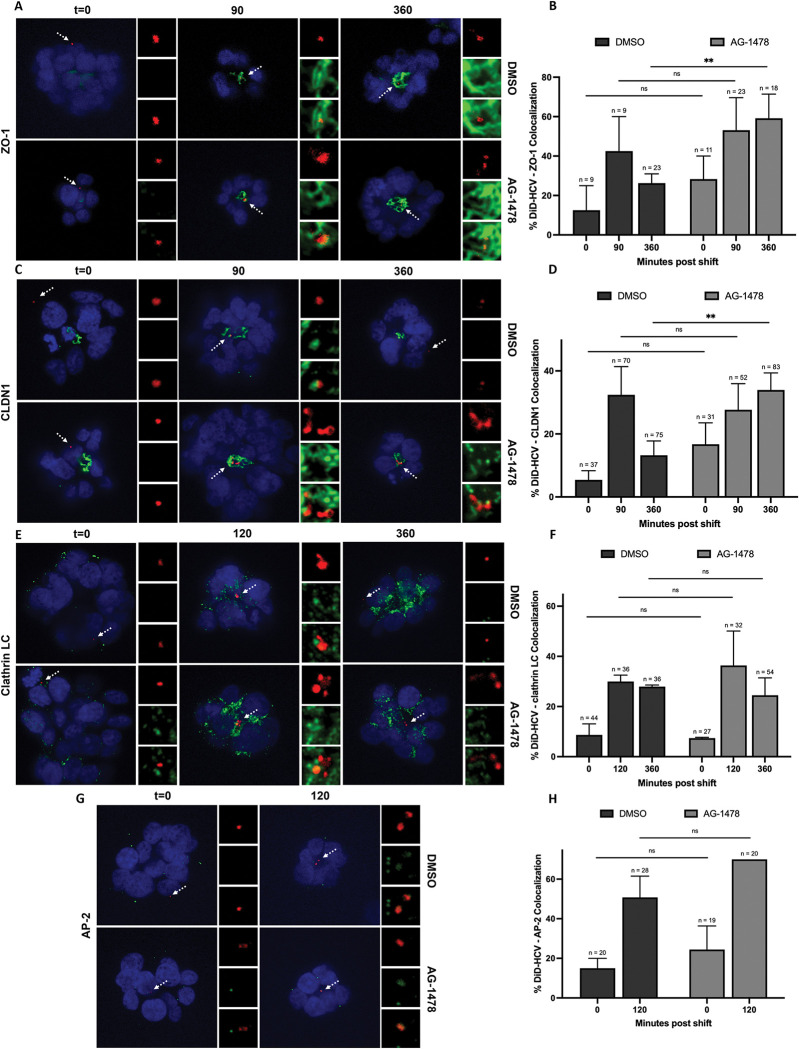
EGFR phosphorylation regulates HCV internalization but is not required for the recruitment of clathrin and AP-2μ1. (A, C, E, and G) Huh-7.5 spheroids were incubated with DMSO or 5 μM AG-1478 for 2 hr, infected with DiD-HCV (red) with DMSO or AG-1478 for 1 hr at 4°C, shifted to 37°C for the indicated times, fixed, and probed for ZO-1 (A), CLDN1 (C), clathrin light chain (clathrin LC) (E) or AP-2μ1 (G) (green). (B, D, F, and H) Quantitation of (A), (C), (E) and (G), respectively. n = total DiD signal. Mean +/- SEM. **p < 0.01.

We previously used single particle tracking to characterize various stages of HCV entry. Over a time course of infection, DiD-HCV initially localizes at the outer basolateral membrane, accumulates at the tight junction peaking around 90 minutes and then decreases colocalization with tight junction markers at 360 minutes, coincident with endocytosis and uncoating of virions. Huh-7.5 spheroids depleted for EGFR accumulated DiD-HCV at the tight junction at both 90 and 360 minutes, indicating a defect in endocytosis. This was associated with a defect in the recruitment of the clathrin machinery (clathrin light chain) [24]. In this study, we tested if endocytosis was dependent on EGFR phosphorylation using EGFR kinase inhibitor AG-1478. We examined the effect of AG-1478 on DiD-HCV colocalization with tight junction markers ZO-1 and CLDN1 respectively. AG-1478 caused the accumulation of DiD-HCV at the tight junction at both 90 and 360 minutes, suggesting that it inhibited HCV internalization (Figs 2A–2D, S2A and S2B).

**Fig 3:**
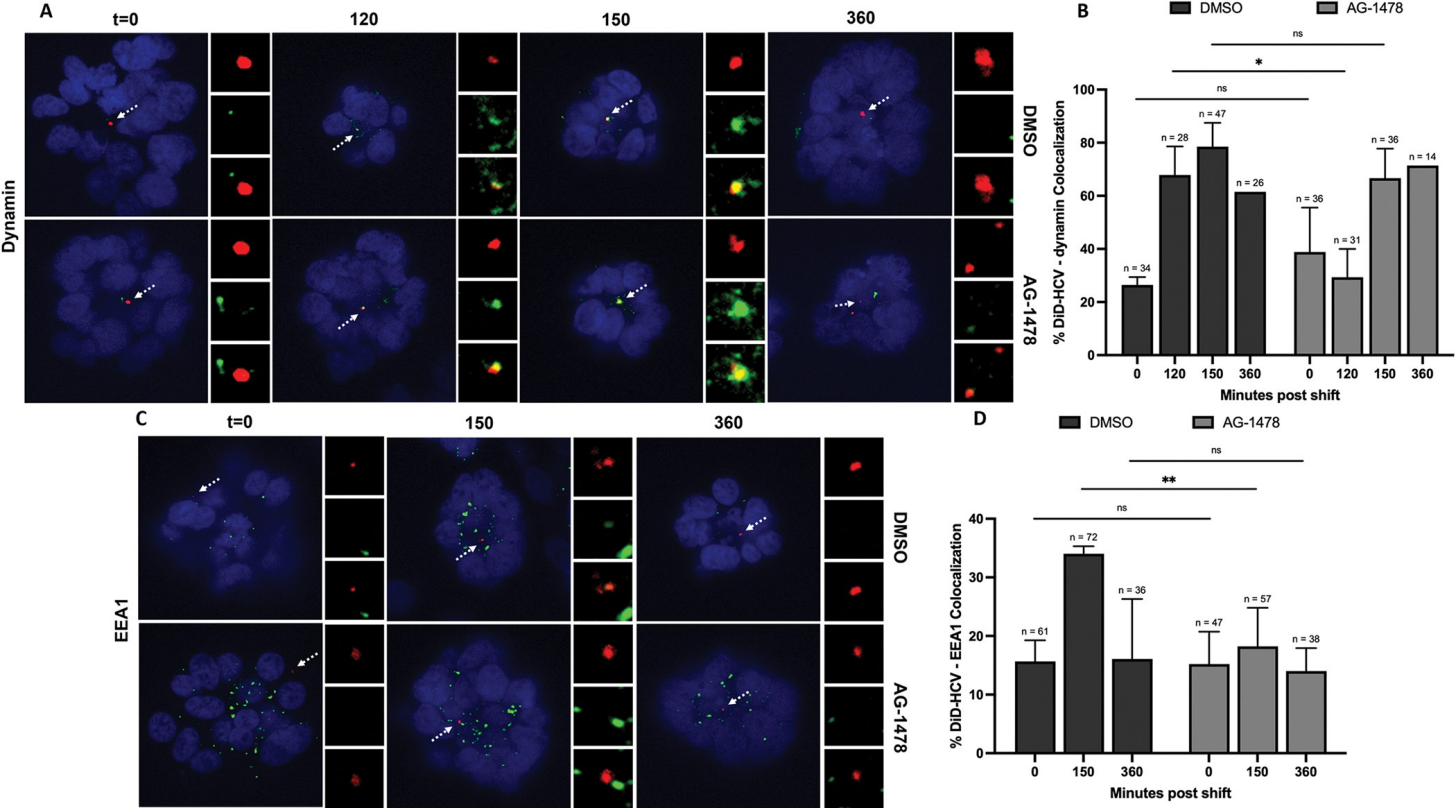
EGFR phosphorylation regulates HCV accumulation in early endosomes. (A and C) Huh-7.5 spheroids were incubated with DMSO or 5 μM AG-1478 for 2 hr, infected with DiD-HCV (red) with DMSO or AG-1478 for 1 hr at 4°C, shifted to 37°C for the indicated times, fixed, and probed for dynamin (A) or EEA1 (C) (green). (B and D) Quantitation of (A) and (C), respectively. n = total DiD signal. Mean +/- SEM. *p < 0.05, **p < 0.01.

We next investigated if the defect in internalization was caused by an inhibition of the recruitment of the clathrin endocytic proteins. We infected DMSO- or AG-1478-treated spheroids with DiD-HCV and probed for clathrin light chain (LC), AP-2μ1, and dynamin, respectively. AG-1478 had no significant effect on DiD-HCV colocalization with clathrin LC or AP-2μ1 over the time course of infection. (Figs 2E–2H, S2C and S2D) We did, however, observe a decrease in the kinetics of dynamin recruitment to HCV virions in the presence of AG-1478. In DMSO-treated spheroids, DiD-HCV/dynamin colocalization increased to 65% at 120 min post temperature shift. In AG-1478-treated spheroids, the kinetics of dynamin localization with DiD-HCV was significantly delayed, with an increase observed at 150, but not 120, minutes (Figs 3A, 3B and S2E).

We then examined the effect of AG-1478 on DiD-HCV colocalization with the early endosomal marker EEA1. AG-1478 treatment blocked the accumulation of DiD-HCV with EEA1, suggesting that it inhibited the accumulation of virions in early endosomes (Figs 3C, 3D and S2F), which are the proposed site of HCV uncoating [31].

### EGFR-mediated RAF-MEK-ERK pathway regulates the sorting of HCV into APPL1- and EEA1-associated early endosomes

**Fig 4:**
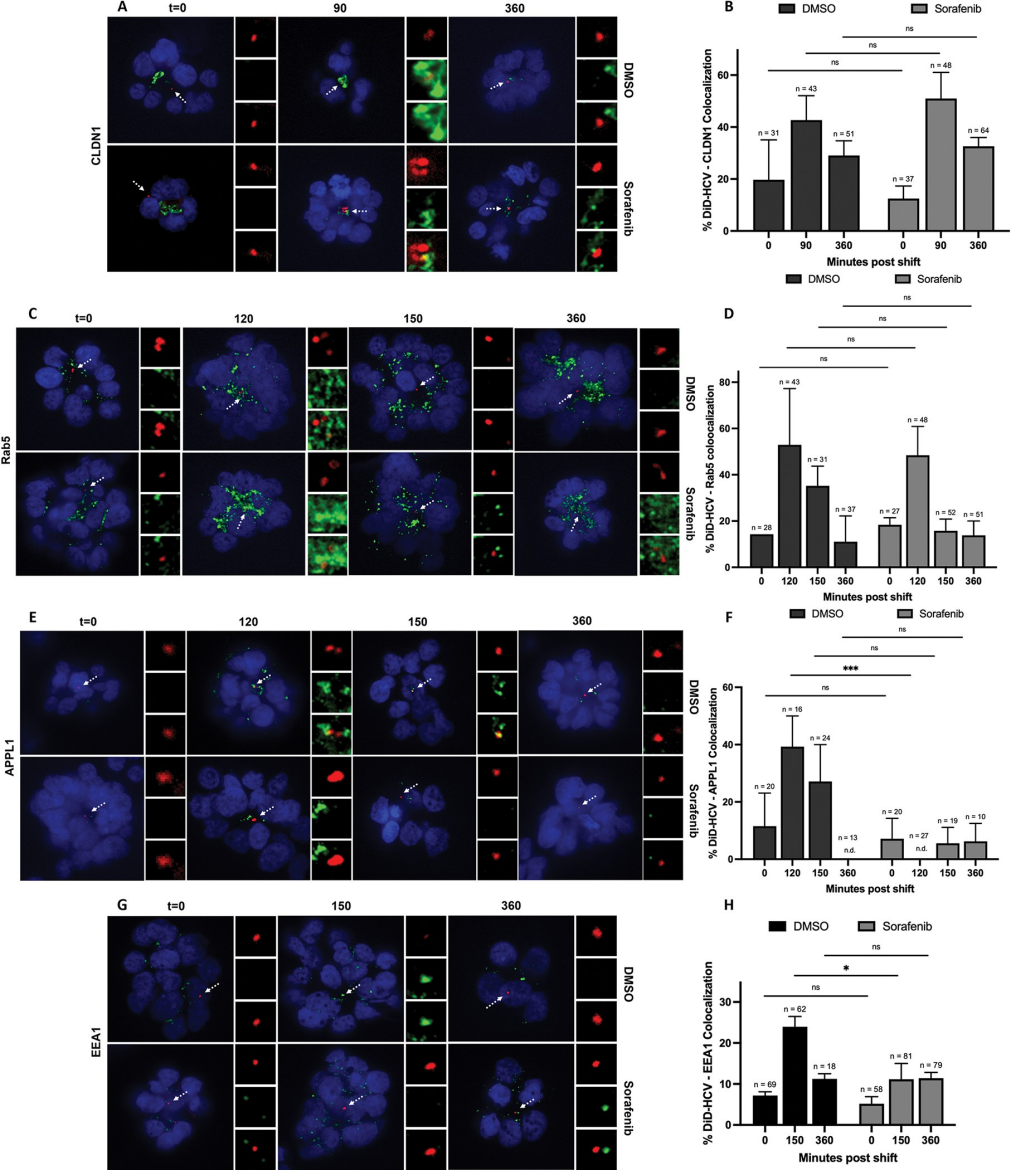
The RAF-MEK-ERK pathway regulates the sorting of HCV into APPL1 and EEA1-associated early endosomes. (A, C, E, and G) Huh-7.5 spheroids were incubated with DMSO or 5 μM sorafenib for 2 hr, infected with DiD-HCV (red) with DMSO or sorafenib for 1 hr at 4°C, shifted to 37°C for the indicated times, fixed, and probed for CLDN1 (A), Rab5 (C), APPL1 (E) or EEA1 (G) (green). (B, D, F, and H) Quantitation of (A), (C), (E) and (G), respectively. n = total DiD signal. Mean +/- SEM. *p < 0.05, ***p < 0.001.

EGFR phosphorylation activates multiple signaling pathways, including RAF-MEK-ERK. We next investigated if the RAF-MEK-ERK pathway regulated HCV internalization. We examined the effect of sorafenib on DiD-HCV colocalization with tight junction markers CLDN1 and ZO-1 respectively. In DMSO- or sorafenib-treated spheroids, the colocalization peaked at 90 min and then decreased at 360 min post temperature shift (Figs 4A, 4B, S3A, S4A and S4B). This indicates that most DiD-HCV particles had undergone internalization, suggesting that HCV internalization is independent of the activation of the RAF-MEK-ERK pathway.

We then examined the effect of sorafenib on DiD-HCV colocalization with markers of the endocytic pathway: Rab5, APPL1, and EEA1. Rab5 is present in both clathrin-coated vesicles and early endosomes [45–48], while APPL1 and EEA1 are Rab5 effector proteins that label two distinct pools of early endosomes [48–51]. During endocytosis, cargos from clathrin-coated vesicles are sequentially sorted into Rab5- and APPL1-positive endocytic organelles then into EEA1-positive early endosomes [47,50,52]. Sorafenib had no effect on DiD-HCV colocalization with Rab5 (Figs 4C, 4D and S3B). In contrast, sorafenib significantly reduced DiD-HCV colocalization with APPL1 and EEA1 (Figs 4E–4H, S3C and S3D). These results suggests that the RAF-MEK-ERK pathway regulates DiD-HCV sorting from clathrin-coated vesicles into APPL1- and EEA1-positive early endosomes. In summary, EGFR recruits clathrin to DiD-HCV in a phosphorylation-independent manner [24]. EGFR phosphorylation is required for DiD-HCV internalization, while EGFR-mediated Raf-MEK-ERK activation is required for the maturation of DiD-HCV-Rab5-positive endocytic vesicles into early endosomes.

### CLDN1 regulates HCV accumulation at the tight junction

**Fig 5:**
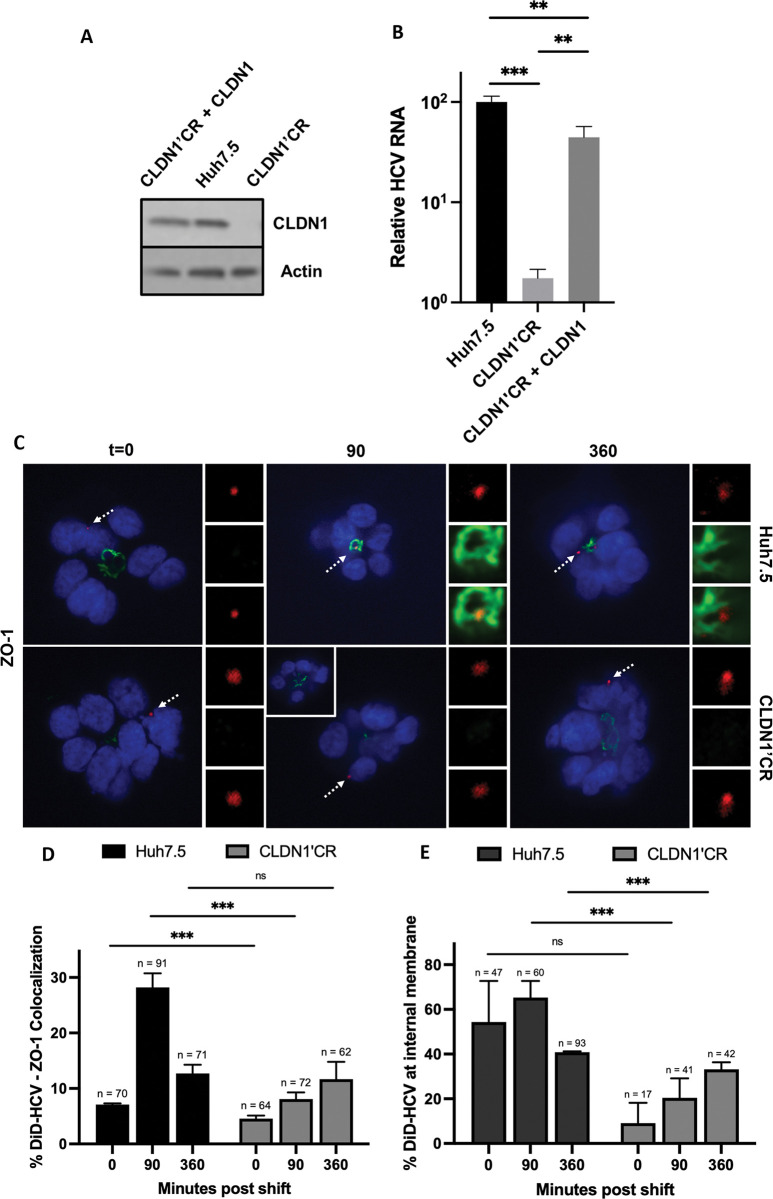
CLDN1 regulates HCV accumulation at the tight junction. (A) Western blot of Huh-7.5 wildtype, CLDN1 CRISPR’ed, and complemented cells. (B) Huh-7.5 wildtype, CLDN1 CRISPR’ed, or complemented cells were seeded onto 96-well plates, infected with HCV for 48 hr, and then analyzed for relative HCV RNA levels. Mean +/- SD. (C) Spheroids of Huh-7.5 wildtype, CLDN1 CRISPR’ed, or complemented cells were infected with DiD-HCV (red) for 1 hr at 4°C, shifted to 37°C for the indicated times, fixed, and probed for ZO-1 (green). (D and E) Quantitation of (C). n = total DiD signal. Mean +/- SEM. *p < 0.05, **p < 0.01, ***p < 0.001.

The roles of tight junction proteins CLDN1 and OCLN had not been studied in the context of polarized hepatoma spheroids. We used CRISPR/Cas9 to knock out CLDN1 in the Huh-7.5 cell line (CLDN1’CR). The CLDN1’CR cell line was then virally transduced to express CLDN1 (CLDN1’CR + CLDN1). (Fig 5A) HCV RNA replication was significantly reduced in CLDN1’CR cells upon HCV infection. The defect was rescued in CLDN1’CR + CLDN1 cells. (Fig 5B). We then investigated the function of CLDN1 in HCV entry. We infected Huh-7.5 or CLDN1’CR spheroids with DiD-HCV and examined DiD-HCV/ZO-1 colocalization. In CLDN1’CR spheroids, the colocalization was significantly lower than that in Huh-7.5 spheroids at 90 min post temperature shift. (Fig 5C and 5D) Significantly fewer DiD-HCV particles accumulated at the internal membranes of CLDN1’CR spheroids (less than 20%) than that of Huh-7.5 spheroids (60%) at 90 min post temperature shift (Fig 5E). The data indicates that CLDN1 is required for efficient DiD-HCV accumulation at the tight junction and subsequent internalization.

### OCLN regulates HCV internalization

**Fig 6:**
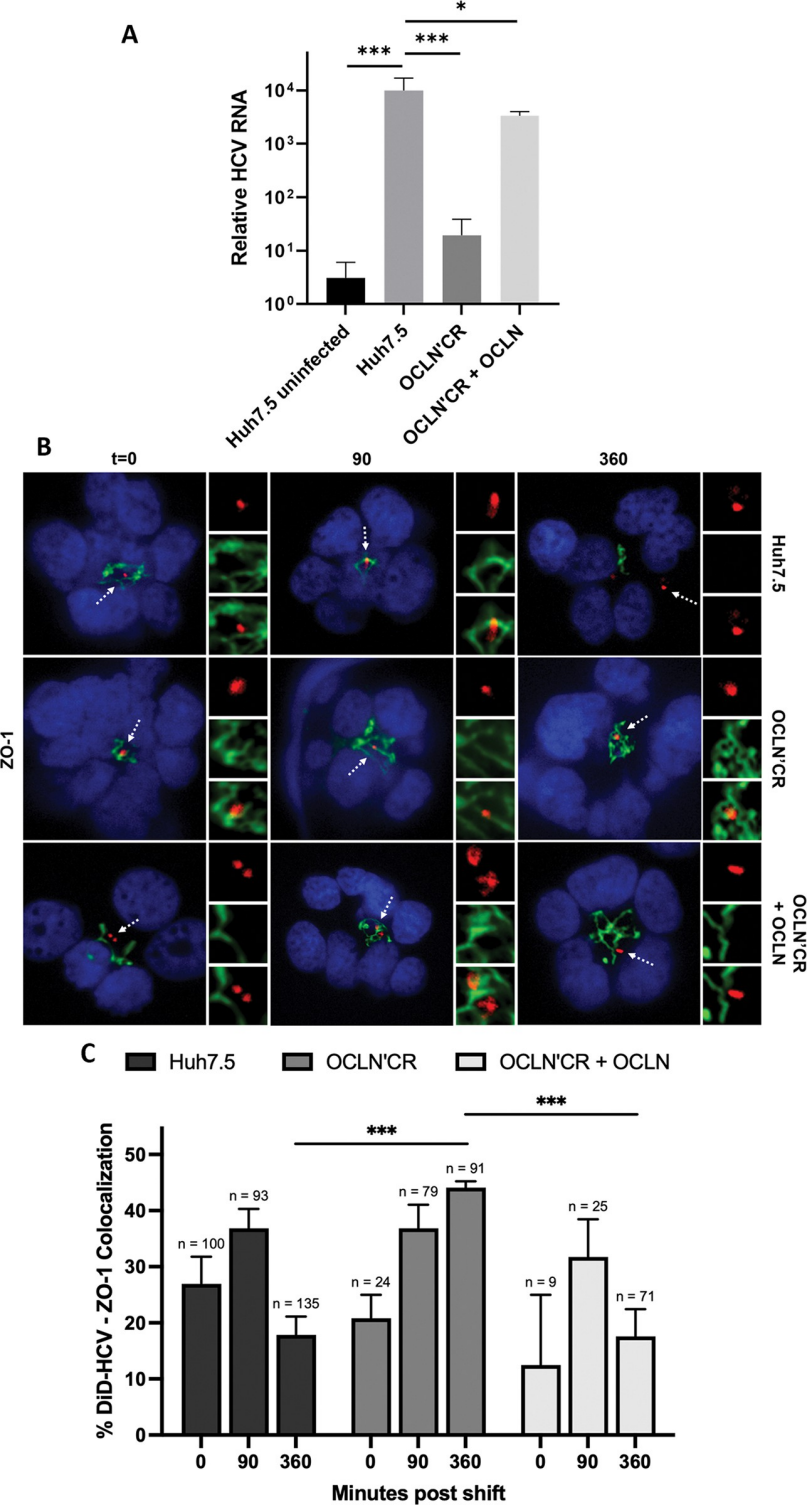
OCLN regulates HCV internalization. (A) Huh-7.5 wildtype, OCLN CRISPR’ed, or complemented cells were seeded onto 96-well plates, infected with HCV for 48 hr, and then analyzed for relative HCV RNA levels. Mean +/- SD. (B) Spheroids of Huh-7.5 wildtype, OCLN CRISPR’ed, or complemented cells were infected with DiD-HCV (red) for 1 hr at 4°C, shifted to 37°C for the indicated times, fixed, and probed for ZO-1 (green). (C) Quantitation of (B). n = total DiD signal. Mean +/- SEM. *p < 0.05, **p < 0.01, ***p < 0.001.

We next generated a Huh-7.5 cell line knocked out of OCLN using CRISPR/Cas9 (OCLN’CR). The OCLN’CR cell line were virally transduced to express OCLN (OCLN’CR + OCLN) (S5A Fig) or Venus-OCLN (S5C Fig). HCV RNA replication was significantly reduced in OCLN’CR cells upon HCV infection. The defect was rescued in complemented cells. (Figs 6A, S5B and S5D) We then investigated the function of OCLN in HCV entry. We infected Huh-7.5 or OCLN’CR spheroids with DiD-HCV and examined DiD-HCV/ZO-1 colocalization. In all the spheroids, the colocalization peaked at 90 min post temperature shift. This indicates that OCLN is not required for DiD-HCV migration to the tight junction. At 360 min post temperature shift, DiD-HCV/ZO-1 colocalization decreased in Huh-7.5 spheroids. This indicates that most DiD-HCV particles had undergone internalization. In contrast, in OCLN’CR spheroids, the colocalization remained high at 360 minutes, indicating that DiD-HCV remained at the tight junction and failed to internalize. (Fig 6B and 6C)

**Fig 7:**
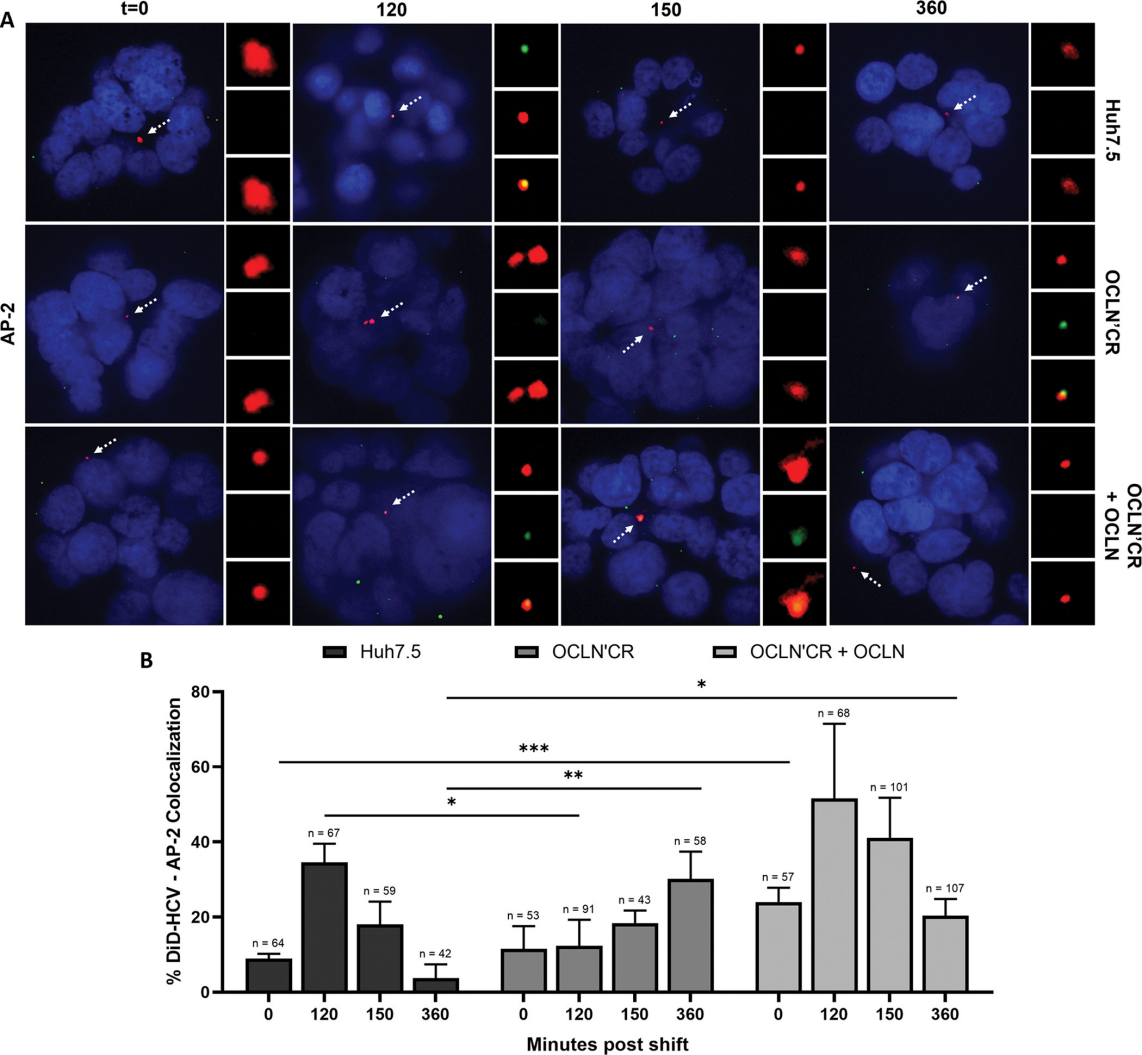
HCV colocalizes with AP-2μ1 in OCLN CRISPR’ed oragnoids. (A) Spheroids of Huh-7.5 wildtype, OCLN CRISPR’ed, or complemented cells were infected with DiD-HCV (red) for 1 hr at 4°C, shifted to 37°C for the indicated times, fixed, and probed for AP-2μ1 (green). (B) Quantitation of (A). n = total DiD signal. Mean +/- SEM. *p < 0.05, **p < 0.01, ***p < 0.001.

We previously showed that DiD-HCV entered Huh-7.5 spheroids via clathrin-mediated endocytosis [24]. We asked whether OCLN was required for the recruitment of the clathrin endocytic machinery to DiD-HCV. Huh-7.5 or OCLN’CR spheroids were infected with DiD-HCV and probed for clathrin LC, AP-2μ1, and dynamin, respectively. In OCLN’CR spheroids, DiD-HCV colocalized with clathrin LC and dynamin over a time course indistinguishably from Huh-7.5 spheroids. (S6A–S6D Fig) For AP-2μ1, in Huh-7.5 or OCLN’CR + OCLN spheroids, DiD-HCV/AP-2μ1 colocalization peaked at 120 min and decreased gradually from 120 min to 360 min post temperature shift. In OCLN’CR spheroids, the kinetics of AP-2μ1 localization with DiD-HCV was significantly delayed. The level of DiD-HCV/AP-2μ1 colocalization in OCLN’CR spheroids at 360 min was comparable to the level in Huh-7.5 spheroids at 120 min. (Fig 7A and 7B)

AP-2μ1 is a subunit of the AP-2 complex which regulates the selection of endocytic cargoes into vesicles. AP-2μ1 directly binds to tyrosine-based sorting signal motifs of cargoes (YXXΦ; Φ: a bulky hydrophobic residue L/I/M/V/F) [53]. Fredriksson et al. [54] suggests that OCLN interacts with AP-2. Moreover, OCLN has two potential YXXΦ motifs: YLSV (aa 172–175) and YNRL (aa 481–484) [55]. However, their functions in endocytosis of OCLN have not been studied. We asked if the motifs were required for HCV infection. For each of the motifs, we mutagenized the tyrosine to phenylalanine and the Φ to hydrophilic threonine. We transduced the OCLN’CR cell line to express OCLN mutated at one (SS1 and SS2) or both (SS1&2) of the motifs. All mutants rescued the defect in HCV RNA replication of the OCLN’CR cell line. (S5A and S5B Fig) This suggests that the motifs are not required for HCV infection and that perhaps OCLN regulates AP-2 recruitment to HCV virions indirectly.

Given the requirement of EGFR phosphorylation for HCV endocytosis, we examined if OCLN was required for the activation of the RAF-MEK-ERK pathway upon HCV entry. We infected Huh-7.5 or OCLN’CR spheroids and lysed them at 120 min post temperature shift. HCV infection induced ERK phosphorylation at T202/Y204 in OCLN’CR spheroids (S5E Fig), indicating that OCLN is not required for HCV-mediated activation of the EGFR-dependent RAF-MEK-ERK pathway.

## Discussion

HCV entry is a multi-step process involving various host factors. The requirement of a broad range of host factors suggests that each performs distinct functions. In our previous work, we developed single particle imaging of HCV in three-dimensional polarized hepatoma spheroids. That study revealed the sequential steps of entry and the functions of entry factors in cell culture that physiologically resembles the polarity of hepatocytes in vivo [24]. In this paper, we performed the assay to study the functions of three crucial host factors in HCV entry: epidermal growth factor receptor (EGFR) and tight junction proteins claudin-1 (CLDN1) and occludin (OCLN).

Despite extensive studies of EGFR, the complex regulations and wide-ranging functions of the receptor are far from fully understood. We demonstrated that EGFR regulation of HCV entry does not solely depend on phosphorylation. shRNA silencing of EGFR inhibited HCV recruitment of clathrin for internalization [24]. When EGFR phosphorylation was blocked by AG-1478, DiD-HCV efficiently recruited clathrin and AP-2 (Fig 2E–2H). The combined results of our two studies suggest that during HCV entry, EGFR regulates the recruitment of clathrin in a phosphorylation-independent manner.

AG-1478 has been shown to inhibit the replication of subgenomic HCV replicons of genotype 1b but not that of 2a expressed in Huh-7 cells [56]. In our study, we observed that AG-1478 inhibited HCV replication in Huh-7.5 cells upon infection with cell culture derived HCV (Fig 1C). Importantly, when HCV endocytosis was bypassed via electroporation of HCV RNA, AG-1478 had no effect on infectious virus production. This suggests that that the antiviral effect of AG-1478 was due to an inhibition of HCV entry but not later stages of the viral life cycle (S1C Fig).

Various mechanisms have been proposed to regulate the recruitment of clathrin to EGFR to initiate endocytosis, such as ubiquitination [57,58] and AP-2 binding sites [59,60]. EGFR is ubiquitinated by E3 ligase c-Cbl via interaction with phosphorylated Y1045 of EGFR [61]. Y1045 is not required for HCV infection, suggestion that its c-Cbl interactions is not required for HCV entry [24]. Moreover, AG-1478 inhibits phosphorylation and hence ubiquitination of EGFR [62]. Therefore, our data suggests that EGFR ubiquitination is not required for HCV recruitment of clathrin (Fig 2E and 2F). AP-2 binding sites and/or other unknown mechanisms may contribute to clathrin recruitment to HCV virions. Moreover, ubiquitination and AP-2 binding sites of EGFR are redundant for EGF endocytosis, which may also be the case for HCV entry [62]. Thus, HCV may utilize multiple functions of EGFR to recruit clathrin.

Despite the recruitment of clathrin and AP-2 to virions (Fig 2E–2H), HCV failed to dissociate from the tight junction and undergo internalization when EGFR phosphorylation was blocked by AG-1478 (Fig 2A–2D). Inhibition of EGFR phosphorylation delayed HCV recruitment of dynamin (Fig 3A and 3B). We interpret the data to suggest that the delayed recruitment of dynamin results in inefficient HCV internalization. EGFR phosphorylation is not required for EGF internalization [63,64]. AG-1478 does not affect the internalization of EGF or EGFR [36,37]. Our data suggests that the internalization of HCV does not fully resemble that of EGF. HCV requires EGFR phosphorylation-dependent functions for efficient internalization. It is independent of the activation of the RAF-MEK-ERK signaling pathway (Figs 4A, 4B, S4A and S4B).

We showed that HCV activates the EGFR-mediated RAF-MEK-ERK signaling pathway for sorting virions into early endosomes after endocytosis. When RAF was inhibited by sorafenib, DiD-HCV was internalized (Figs 4A, 4B, S4A and S4B) and sorted into Rab5-positive early endocytic compartments (Fig 4C and 4D). However, DiD-HCV failed to colocalize with APPL1 or EEA1 when RAF was inhibited (Fig 4E–4H). Both are effectors of Rab5 and preferentially interact with the active form of Rab5 (Rab5-GTP) [49–51,65,66]. The interaction regulates the localization of APPL1 on membranes [50] and the maturation and formation of EEA1-positive endosomes [65]. EGFR-natural ligand EGF activates Rab5 via EGFR phosphorylation [66]. Therefore, we propose that HCV-activated RAF-MEK-ERK pathway facilitates Rab5 activation and hence the fusion or maturation of HCV-containing vesicles to early endosomes. RAF or proteins downstream of RAF in the pathway are potential regulators of Rab5. EGFR phosphorylation regulates the fate of internalized receptor. Phosphorylated EGFR is preferentially sorted to endosomes then lysosomes for degradation, rather than recycling back to the plasma membrane [63,64]. Our finding suggests that HCV hijacks this EGFR function in cargo endocytic sorting and uncoats prior to lysosomal degradation.

EGFR and downstream signaling pathways are crucial for the entry of a diverse range of viruses [67,68]. Macropinocytosis of respiratory syncytial virus is regulated by the PI3K-AKT and PKC pathways downstream of EGFR [69]. Similarly, vaccinia virus is internalized via EGFR-mediated macropinocytosis [70]. EGFR kinase activity regulates clathrin-mediated endocytosis of hepatitis B virus independent of the RAF-MEK-ERK signaling pathway [71,72]. MEK is required for clathrin-independent internalization of herpes simplex virus 1 [73]. Our findings demonstrate for the first time EGFR-RAF-MEK-ERK-mediated sorting of endocytosed virions into early endosomes for fusion. We also revealed that HCV utilizes multiple functions of EGFR across sequential steps of virion internalization. A recent study showed that RAF is required for clathrin-mediated endocytosis of influenza A virus (IAV) [74]. Whether it regulates the sorting of IAV into endosomes is an open question.

Furthermore, we found that claudin-1 (CLDN1), a tight junction protein that is essential for HCV entry [13], is required for HCV accumulation at the tight junction (Fig 5C–5E). This result was somewhat unexpected because, in polarized hepatoma spheroids, CLDN1 does not have direct contact with basolateral virions. Knocking out CLDN1 did not obviously affect the polarization of spheroids, or the integrity of the tight junction (Fig 5C). Li et al. [75] showed that siRNA silencing of CLDN1 did not affect the expression or tight junction localization of E-cadherin, an adhesion protein. Therefore, the defect in HCV entry was not due to an obvious change in cell structure. CLDN1 interacts with CD81 and the interaction is required for HCV entry [16,19,20,76]. CD81 regulates HCV trafficking to the tight junction in polarized hepatoma spheroids [24]. We envision two plausible scenarios. The CLDN1-CD81 interaction might serve as an anchor to tether migrating HCV virions to the tight junction. In the absence of CLDN1, migrating HCV might resurface at the basolateral membrane. Alternatively, CLDN1 may be required for signaling events that promote the CD81-driven migration to the tight junction. Future studies will attempt to unravel that mystery.

We found that the tight junction protein occludin (OCLN) is required for HCV internalization (Fig 6B and 6C). It was proposed that OCLN interacted with AP-2 via the tyrosine-based sorting signal motifs in the cytosolic tail of OCLN for internalization [54,55]. We showed that the sorting signal motifs of OCLN are not required for HCV endocytosis (S5A and S5B Figs). However, we did observe a significantly decreased kinetics of AP-2 recruitment to HCV virions in the absence on OCLN (Fig 7A and 7B). Our finding suggests that while OCLN AP-2 binding motifs are not required for HCV entry, OCLN contributes indirectly to efficient AP-2 recruitment. Without OCLN, HCV colocalized with clathrin and dynamin (S6A–S6D Fig). Uninternalized HCV were retained at the tight junction (Fig 6B and 6C) and accumulated AP-2 (Fig 7A and 7B). OCLN appears to promote the stabilization of clathrin-coated pits for successful endocytosis [77].

Huntingtin-interacting protein 1 (HIP1) and Huntingtin-interacting protein 1-related (HIP1R) are components of clathrin coated pits. They bind differentially to actin and endocytic proteins including clathrin light chain and AP-2. [78,79] The interactions facilitate clathrin assembly [79] and hence regulate endocytosis of membrane proteins such as EGFR [80,81]. Upon HCV entry, HIP1R is recruited to CD81 [82]. siRNA silencing of HIP1 and HIP1R respectively inhibited HCV infection [32]. Potential interaction of EGFR or OCLN with HIP1/HIP1R during HCV internalization will be addressed in future studies.

**Fig 8:**
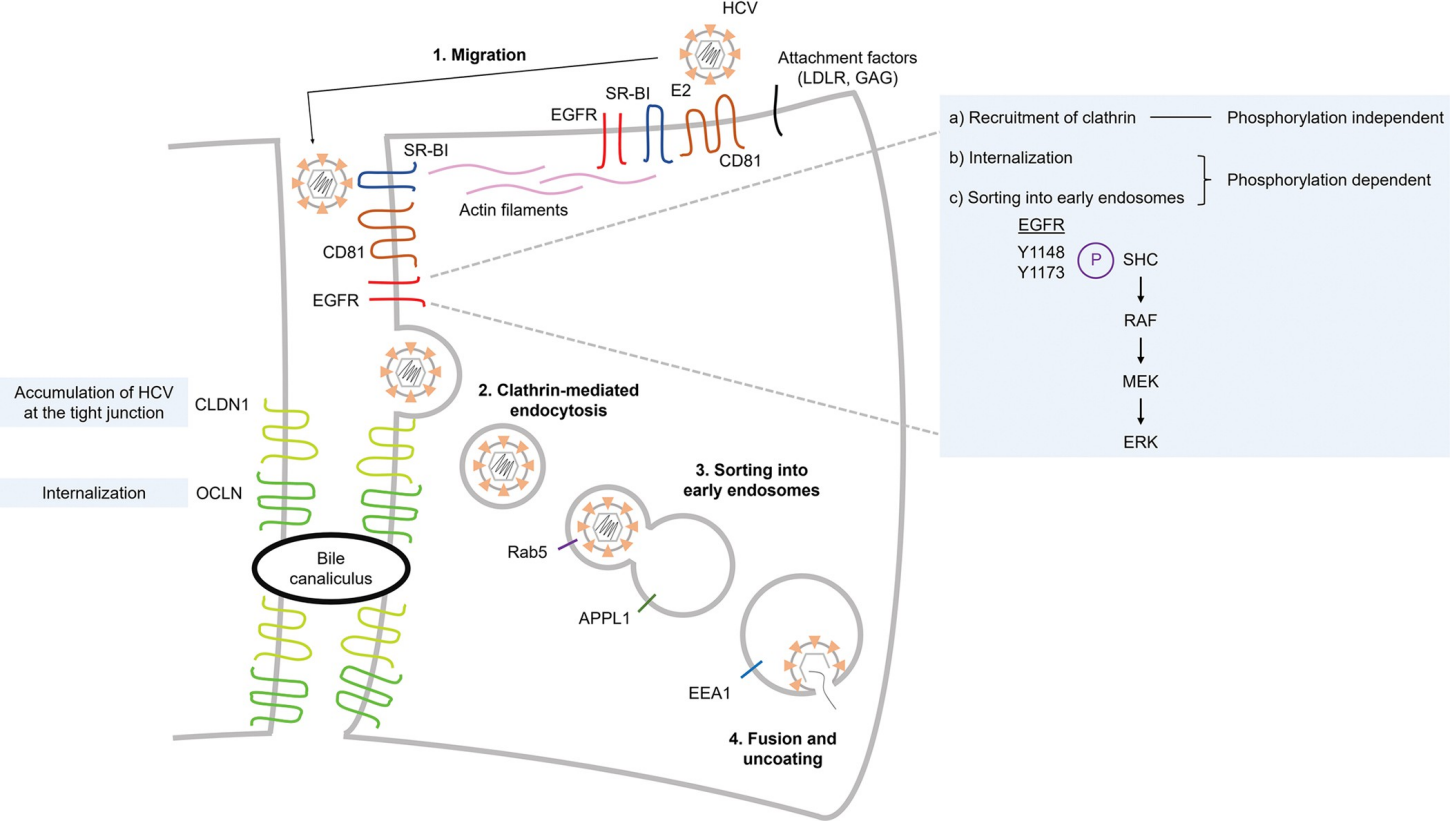
Model of HCV entry into polarized hepatoma spheroids.

Here, we propose a further refined model of HCV entry into polarized hepatoma spheroids (Fig 8). There are sequential events: (1) HCV virions engage CD81, then SR-B1 in a complex containing EGFR and migrate from the basolateral membrane to the tight junction via actin [24]. (2) In a process that requires CLDN1, HCV virions accumulate at the tight junction. (3) Once at the tight junction, HCV virions recruit clathrin in an EGFR phosphorylation-independent manner. (4) HCV is then internalized in a process that requires both EGFR phosphorylation and OCLN. (5) HCV entry activates the RAF-MEK-ERK signaling pathway downstream of EGFR phosphorylation. This pathway regulates the sorting and maturation of internalized HCV into APPL1- and EEA1-associated early endosomes for subsequent virion fusion and uncoating. Future studies will investigate the mechanism behind the requirement of CLDN1 for HCV accumulation at the tight junction and the roles of OCLN for the clathrin-mediated endocytosis of HCV.

## Materials and methods

### Cell culture

Huh-7.5 cells [83] were cultured in Dulbecco’s modified high glucose media (DMEM; Gibco) supplemented with 10% fetal bovine serum (FBS; Gemini), 0.1 mM nonessential amino acids (NEAA; Gibco), 1% penicillin-streptomycin (Millipore Sigma), and incubated at 37°C in 5% CO_2_. Spheroids were cultured as described [24]. Briefly, Huh-7.5 cells were trypsinized and diluted in DMEM + 10% FBS to a final concentration of 1 x 10^5^ cells/mL. Equal volumes of diluted cells and Matrigel (Growth factor reduced, phenol red-free; Corning) were mixed and seeded onto cover glasses (ThermoFisher) or glass dishes (Ibidi). Cells were cultured for 6–8 days, changing media every other day.

### CLDN1 knockout and complemented cell lines

Huh-7.5 cells were transfected with MISSION CRISPR gRNA (ID: HSPD0000052528, vector: LV05, gRNA sequence 5’-ATA CAC TTC ATG CCA ACG G, Millipore Sigma) using Lipofectamine 2000 Transfection Reagent (Invitrogen). Transfected cells were selected using puromycin (3 μg/mL, Gibco). Cell clones were then isolated and analyzed for protein expression via immunoblot. To create CLDN1 expressing construct, CLDN1 ORF (template: OHu20823D, GenScript) was amplified using: forward primer (5’- GGA TCT ATT TCC GGT GAA TTC ATG GCC AAC GCG GGG CTG) and reverse primer (5’- ATC CGC GGC CGC TCT AGA TCA CAC ACG TAG TCT TTC CCG CTG GAA GG). pLVX vector was digested with EcoRI and XbaI. Amplified fragments were then inserted into digested pLVX with In-Fusion HD Cloning Plus (Takara) per the manufacturer’s instructions.

### OCLN knockout and complemented cell lines

OCLN’CR and Venus-OCLN expressing complemented cell lines were gifts of Matthew Evans, Icahn School of Medicine at Mount Sinai. To create OCLN expressing construct, OCLN ORF (template: OHu28110D, GenScript) was amplified using: forward primer (5’-GGA TCT ATT TCC GGT GAA TTC ATG TCA TCC AGG CCT CTT) and reverse primer (5’-ATC CGC GGC CGC TCT AGA CTA TGT TTT CTG TCT ATC ATA GTC TCC). pLVX vector was digested with EcoRI and XbaI. Amplified fragments were then inserted into digested pLVX with In-Fusion HD Cloning Plus (Takara) per the manufacturer’s instructions. To create constructs expressing OCLN mutants, Q5 Site-Directed Mutagenesis Kit (NEB) was used per the manufacturer’s instructions with pLVX OCLN as template and primer sets: 1) SS1 forward: 5’-AGT ACG ATA ATA GTG AGT GCT ATC C; reverse: 5’-TAA GAA GTA TCT TCT TGT TCT GG; 2) SS2 forward: 5’-AGA ACG AAG CAA GTG AAG GGA TC; reverse: 5’-ATT GAA TTC ATC AGC AGC AGC CA.

### Pseudoparticle production and transduction

293T cells were transfected with each of the expression constructs using 2nd Gen Packaging Mix & Lentifectin Combo Pack (abm) per the manufacturer’s instructions. Supernatants were harvested 48 hr after transfection and filtered through a 0.45-micron filter. Huh-7.5 cells were added the supernatants and 8 μg/mL polybrene (Millipore Sigma), spun for 500xg for 1.5 hr at room temperature, and incubated for 4 hr.

### Highly infectious virus preparation

HCV stocks were generated as described [24,32,84]. Briefly, Huh-7.5 cells were electroporated with HCV genotype 2a RNA (infectious clone pJFHxJ6-CNS2C3). Viral supernatants were collected daily for up to 8 days after electroporation, filtered through a 0.22-micron filter, then stored at 4°C. Viral titers were determined by limiting dilution and immunohistochemical staining with a NS5A antibody (9E10) (gift of Charles Rice, Rockefeller University) as described [85]. HCV stocks were concentrated using polyethylene glycol 8000 (PEG; ThermoFisher) as described [24,32,83]. Briefly, viral supernatant was incubated at 4°C overnight with PEG (final concentration 8% (w/v)). Virus was then centrifuged (8000xg, 20 min, 4°C) and pellet was resuspended in 10 mL of the original supernatant. Resuspended sample was centrifuged again (8000xg, 10 min, 4°C) and pellet was resuspended in 1 mL DMEM. 1 mL of concentrated virus was incubated with 5 μl of lipophilic dye DiD (Invitrogen) for 90 min with shaking at 4°C. Labeled virus was loaded onto a 10%–50% (w/v) OptiPrep iodixanol gradient (Millipore Sigma) in sterile water and centrifuged for 32 x 10^6^ revolutions (30,000 RPMs, 18 hr, 4°C). 0.5 mL fractions were isolated from the gradient. Each fraction was analyzed for HCV RNA levels by Trizol-LS extraction followed by quantitative real-time PCR and infectious virus. Fractions with the best specific infectivity were added to Amicon Ultra 100k filters (Millipore Sigma) and centrifuged (14000xg, 30 min, 4°C). Filters were then inverted in a new tube and centrifuged (1000xg, 2 min, 4°C).

### Cell recovery from Matrigel

Huh-7.5 cells were mixed with Matrigel (total volume 500 mL/dish), seeded onto glass dishes (Ibidi), and cultured for 8 days as described above. Cells were serum starved in DMEM + 0% FBS for 10 hr, then treated (if indicated) with 5 μM sorafenib or AG-1478 (Millipore Sigma) in DMEM + 0% FBS for 2 hr. Cells were then infected with PEG-concentrated HCV (MOI = 4) and sorafenib or AG-1478 (if indicated), incubated on ice for 1 hr, then incubated in 37°C incubator. 1 hr prior to lysis, cells were added 500 mL cell recovery solution (Corning), shaken for 35 min at 4°C, then centrifuged (1200xg, 5 min, 4°C). Pellet was washed twice in PBS. After the last wash, pellet was lysed. For mock infection, medium supernatant was collected from Huh-7.5 cells, filtered, then PEG precipitated as described above.

### Western blot analysis

All cells were lysed in 1% NP40 buffer (150 mM NaCl, 50 mM Tris-HCl pH 8, 10% glycerol, 2 mM EDTA) supplemented with 1 mM cOmplete Mini protease inhibitors (Roche) and 1 mM sodium orthovanadate (ThermoFisher). Proteins were separated on a 4–20% SDS-PAGE gel (BioRad) and transferred to polyvinylidene difluoride (PVDF) membrane. Membrane was incubated in 5% BSA and 0.1% Tween-20 in PBS for 1 hr, incubated overnight at 4°C with primary antibodies (1:10000 anti-β-actin, Santa Cruz #sc-47778; 1:1000 anti-SHC, BD Transduction #610878; 1:1000 anti-ERK, Invitrogen #13–6200; 1:1000 anti-phospho-SHC (Tyr239/240), Cell Signaling #2434; 1:2000 anti-phospho-p44/42 ERK (Thr202/Tyr204), Cell Signaling #4370; 1:1000 anti-EGFR, Cell Signaling #2232; 1:700 anti-phospho-Thr/Tyr, Cell Signaling #9381; 1:500 anti-CLDN1, Invitrogen #37–4900; 1:350 anti-OCLN, Invitrogen #33–1500), then incubated for 1 hr at room temperature with horseradish peroxidase-conjugated secondary antibodies (1:10000 anti-rabbit, Cell Signaling #7074; 1:10000 anti-mouse, Cell Signaling #7076). Membrane was added SuperSignal West Pico PLUS Chemiluminescent or West Femto Maximum Sensitivity substrate (ThermoFisher) and exposed to CL-XPosure film (ThermoFisher).

### Immunofluorescence assays

As described [24]. Briefly, Huh-7.5 cells were mixed with Matrigel (total volume 75 μl/cover glass), seeded onto cover glasses in 24-well plates, and cultured for 7 days. If indicated, cells were treated with 5 μM sorafenib or AG-1478 (Millipore Sigma) in DMEM + 10% FBS 2 hr prior to infection. Cells were pre-chilled on ice for 15 min, infected with DiD-labeled HCV diluted in DMEM + 10% FBS, incubated on ice for 1 hr, then incubated in 37°C incubator (time of temperature shift: t = 0). Cells were fixed in 4% paraformaldehyde (PFA) (20 min), permeabilized with 0.5% Triton x-100 in PBS (10 min), then rinsed with 0.1 M Glycine in PBS (3 times, 10 min each). Cells were incubated for 2 hr at 37°C in blocking solution (0.1% BSA, 0.2% Triton x-100, 0.005% Tween-20, and 20% goat serum in PBS). Cells were incubated overnight at 4°C with primary antibodies (1:450 anti-ZO-1, Invitrogen #18–7430; 1:100 anti-CLDN1, Santa Cruz #sc-81796; 1:200 anti-clathrin LC, Santa Cruz #sc-12735; 1:100 anti-AP-2μ1, Santa Cruz #sc-49150; 1:75 anti-dynamin I/II, Santa Cruz #sc-390160; 1:75 anti-Rab5, Santa Cruz #sc-46692; 1:75 anti-APPL1, Santa Cruz #sc-271909; 1:600 anti-EEA1, Abcam #ab2900) After overnight incubation, the Matrigel was placed for 10 min at room temperature. Cells were washed (3 times, 20 min each) with wash buffer (0.1% BSA, 0.2% Triton x-100, and 0.005% Tween-20 in PBS). Cells were incubated for 1 hr at room temperature with 1:1000 Alexa Fluor-conjugated secondary antibody (488, Invitrogen) in blocking solution, then washed (3 times, 20 min each) with wash buffer. Cover glasses were mounted with ProLong Gold Antifade Mountant with DAPI (Invitrogen).

### Cell viability assay

Cell viability was determined using CellTiter-Glo Luminescent Cell Viability Assay (Promega) per the manufacturer’s instructions.

### Entry bypass assay

Huh-7.5 cells were electroporated with HCV RNA as described above and then cultured with DMEM + 10% FBS. 24 hr post electroporation, medium was replaced with 5 μM sorafenib or AG-1478 (Millipore Sigma) in DMEM + 10% FBS. 48 h post electroporation, viral supernatants were collected, filtered through a 0.22-micron filter, then stored at 4°C. Viral titers were determined as described above.

### HCV RNA quantitation

RNA was extracted using NucleoSpin 96-well kit for RNA purification (Macherey-Nagel). RNA copy number was determined using quantitative real-time PCR as described [86]. Copy numbers of HCV and 18S RNA were determined via comparison to concentration standards. HCV RNA was normalized to 18S RNA of the same sample, then to the normalized DMSO or Huh-7.5 control for relative HCV RNA levels.

### Immunoprecipitation

Unpolarized Huh-7.5 cells were treated with 5 μM AG-1478 (Millipore Sigma) in DMEM + 0% FBS for 2 hr. 40 ng/mL recombinant human EGF (Gibco) was added if indicated. Cells were incubated for 15 min before lysis. Lysates were incubated overnight at 4°C with 2 μg/mL anti-EGFR antibody (Invitrogen, #MA5-13269). Per sample 100 μl Dynabeads M-280 Sheep Anti-Mouse IgG (Invitrogen) was washed four times with PBS and then incubated with the antibody-bound lysates overnight at 4°C. Beads were washed four times with 1% NP40 buffer (described above) supplemented with 1 mM cOmplete Mini protease inhibitors (Roche) and 1 mM sodium orthovanadate (ThermoFisher). After the final wash, beads were boiled for 5 min at 95°C in 4X sample buffer (250 mM Tris-HCl pH 6.8, 8% (w/v) SDS powder, 40% glycerol, 20% B-mercaptoethanol, bromophenol blue).

### Confocal microscopy analysis

As described [24]. Briefly, imaging was performed using Slidebook software and Olympus DSU Spinning Disc Confocal with a 100X NA 1.45 oil-immersion TIRFM objective (intensification: 2, auxiliary: 255). Filter sets used were: DsRed (DiD-HCV), GFP (Alexa Fluor 488), and 405 (DAPI). Z stacks of the spheroids were imaged with a step size of 0.5 μm. Images were analyzed with ImageJ (NIH). DiD puncta were evaluated for their colocalization with indicated antibodies. Exposure time and thresholds were standardized for each experiment set. ‘n = ‘ value was the total number of DiD puncta quantified per treatment.

### Statistical analysis

Data were shown as mean ± standard deviation or mean ± standard error of the mean as indicated in the figure legends. Statistical significance was determined using two-tailed Student’s t test. p values larger than or equal to 0.05 were displayed as ns.

## Supporting information

S1 Fig Inhibitors AG-1478 and sorafenib. (A) Huh-7.5 spheroids were serum starved, incubated with 5 μM AG-1478 or sorafenib for 2 hr if indicated, infected with concentrated HCV with 5 μM AG-1478 or sorafenib for 1 hr at 4°C, shifted to 37°C, processed with Matrigel cell recovery solution, and lysed at 120 min post temperature shift. Lysate samples were immunoblotted for the indicated proteins. (B) Huh-7.5 cells were serum starved, incubated with DMSO or 5 μM AG-1478 for 2 hr, stimulated with 40 ng/mL EGF with DMSO or AG-1478 for 15 min and lysed. EGFR was immunoprecipitated from the lysate samples and immunoblotted for the indicated proteins. (C) Huh-7.5 cells were electroporated with HCV RNA. 24 hr post electroporation, medium was replaced with 5 μM sorafenib or AG-1478 in medium. 48 hr post electroporation, viral supernatants were collected, and infectious viral titers were determined. Mean +/- SD.
(TIF)

S2 Fig AG-1478 does not affect the expression of markers of tight junction, the endocytic pathway, or early endosomes. (A-F) Huh-7.5 spheroids were incubated with DMSO or 5 μM AG-1478 for 2h, infected with DiD-HCV (red) with DMSO or AG-1478 for 1 hr at 4°C, shifted to 37°C for the indicated times, fixed, and probed for ZO-1 (A), CLDN1 (B), clathrin light chain (clathrin LC) (C), AP-2μ1 (D), dynamin (E) or EEA1 (F). Fluorescence intensity was measured. Mean +/- SD.
(TIF)

S3 Fig Sorafenib does not affect the expression of markers of tight junction or early endosomes. (A-D) Huh-7.5 spheroids were incubated with DMSO or 5 μM sorafenib for 2h, infected with DiD-HCV (red) with DMSO or sorafenib for 1 hr at 4°C, shifted to 37°C for the indicated times, fixed, and probed for CLDN1 (A), Rab5 (B), APPL1 (C) or EEA1 (D). Fluorescence intensity was measured. Mean +/- SD.
(TIF)

S4 Fig Inhibition of the RAF-MEK-ERK pathway does not affect HCV internalization. (A) Huh-7.5 spheroids were incubated with DMSO or 5 μM AG-1478 for 2h, infected with DiD-HCV (red) with DMSO or AG-1478 for 1 hr at 4°C, shifted to 37°C for the indicated times, fixed, and probed for ZO-1 (green). (B) Quantitation of (A). n = total DiD signal. Mean +/- SEM.
(TIF)

S5 Fig OCLN CRISPR’ed and complemented cells. (A and C) Western blot of Huh-7.5 wildtype, OCLN CRISPR’ed, and complemented cells. (B and D) Huh-7.5 wildtype, OCLN CRISPR’ed, or complemented cells were seeded onto 96-well plates, infected with HCV for 48 hr, and then analyzed for relative HCV RNA levels. (E) Spheroids of Huh-7.5 wildtype or OCLN CRISPR’ed cells were serum starved, incubated with 5 μM sorafenib (if indicated) for 2 hr, infected with concentrated HCV with sorafenib (if indicated) for 1 hr at 4°C, shifted to 37°C, processed with Matrigel cell recovery solution, and lysed at 120 min post temperature shift. For EGF-treated sample, spheroids of Huh-7.5 wildtype cells were serum starved, stimulated with 40 ng/mL EGF, processed with Matrigel cell recovery solution, and lysed at 60 min post EGF stimulation. Lysate samples were immunoblotted for the indicated proteins. Mean +/- SD. **p < 0.01.
(TIF)

S6 Fig OCLN is not required for the recruitment of clathrin light chain or dynamin to HCV. (A and C) Spheroids of Huh-7.5 wildtype or OCLN CRISPR’ed cells were infected with DiD-HCV (red) for 1 hr at 4°C, shifted to 37°C for the indicated times, fixed, and probed for clathrin light chain (clathrin LC) (A) or dynamin (C) (green). (B and D) Quantitation of (A) and (C), respectively. n = total DiD signal. Mean +/- SEM.
(TIF)
