# Supplementary figures and images for: Roles of epidermal growth factor receptor, claudin-1 and occludin in multi-step entry of hepatitis C virus into polarized hepatoma spheroids
Source: PLoS Pathog. 2023 Dec 29;19(12):e1011887. doi: 10.1371/journal.ppat.1011887 (PMC10756512; doi:10.1371/journal.ppat.1011887)

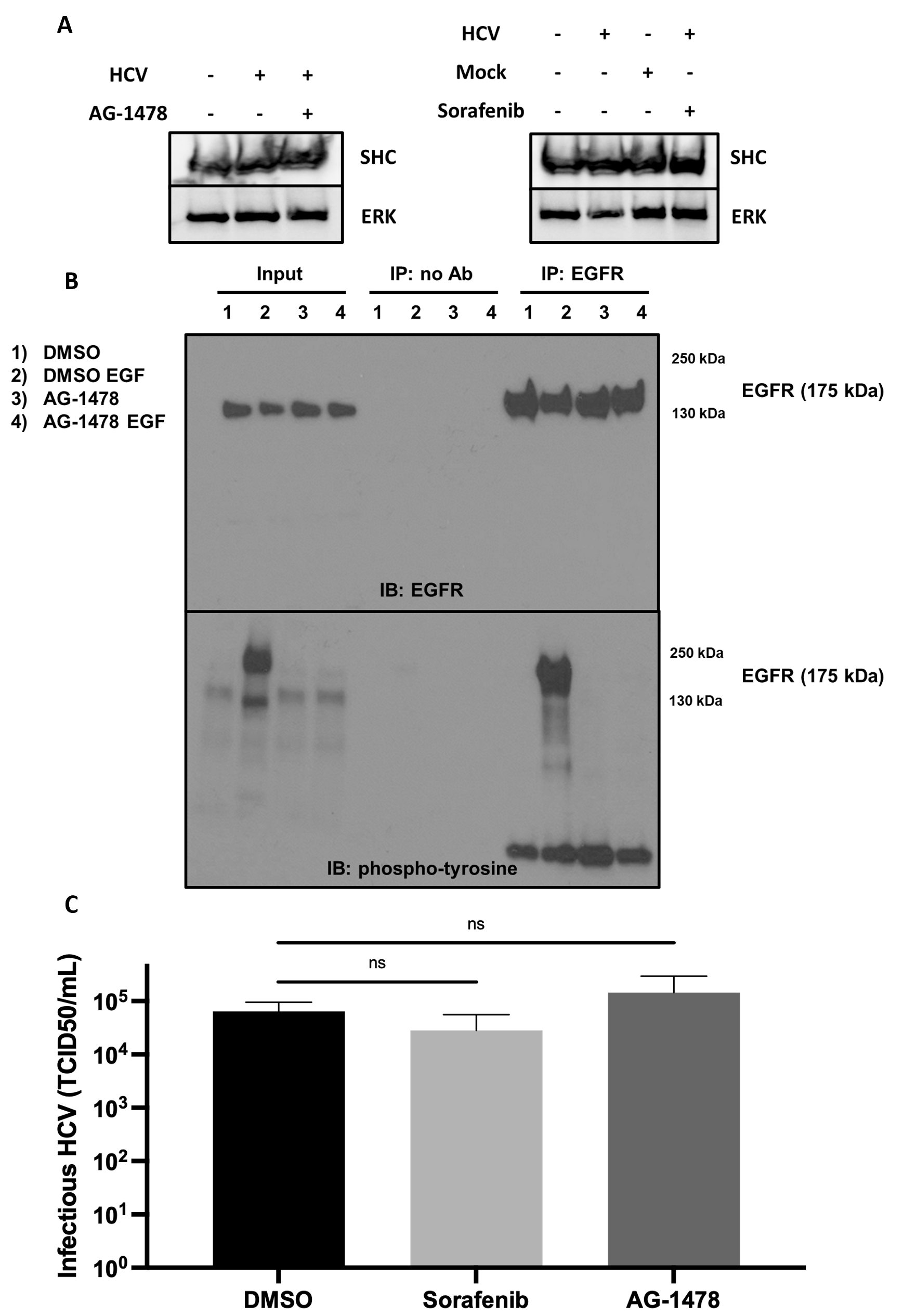

Supplement: S1 Fig — (A) Huh-7.5 spheroids were serum starved, incubated with 5 μM AG-1478 or sorafenib for 2 hr if indicated, infected with concentrated HCV with 5 μM AG-1478 or sorafenib for 1 hr at 4°C, shifted to 37°C, processed with Matrigel cell recovery solution, and lysed at 120 min post temperature shift. Lysate samples were immunoblotted for the indicated proteins. (B) Huh-7.5 cells were serum starved, incubated with DMSO or 5 μM AG-1478 for 2 hr, stimulated with 40 ng/mL EGF with DMSO or AG-1478 for 15 min and lysed. EGFR was immunoprecipitated from the lysate samples and immunoblotted for the indicated proteins. (C) Huh-7.5 cells were electroporated with HCV RNA. 24 hr post electroporation, medium was replaced with 5 μM sorafenib or AG-1478 in medium. 48 hr post electroporation, viral supernatants were collected, and infectious viral titers were determined. Mean +/- SD. (TIF) [file ppat.1011887.s001.tif]

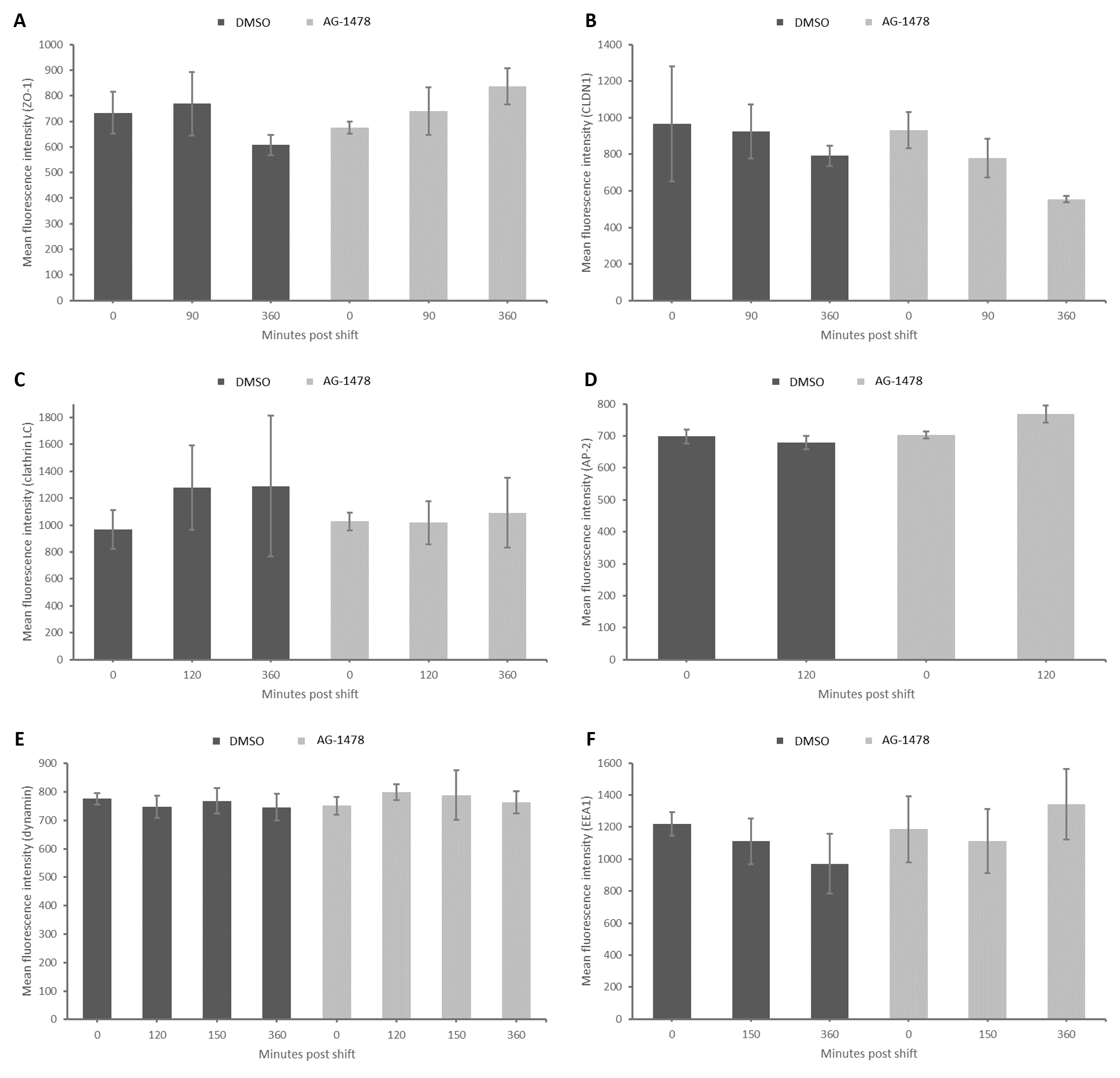

Supplement: S2 Fig — (A-F) Huh-7.5 spheroids were incubated with DMSO or 5 μM AG-1478 for 2h, infected with DiD-HCV (red) with DMSO or AG-1478 for 1 hr at 4°C, shifted to 37°C for the indicated times, fixed, and probed for ZO-1 (A), CLDN1 (B), clathrin light chain (clathrin LC) (C), AP-2μ1 (D), dynamin (E) or EEA1 (F). Fluorescence intensity was measured. Mean +/- SD. (TIF) [file ppat.1011887.s002.tif]

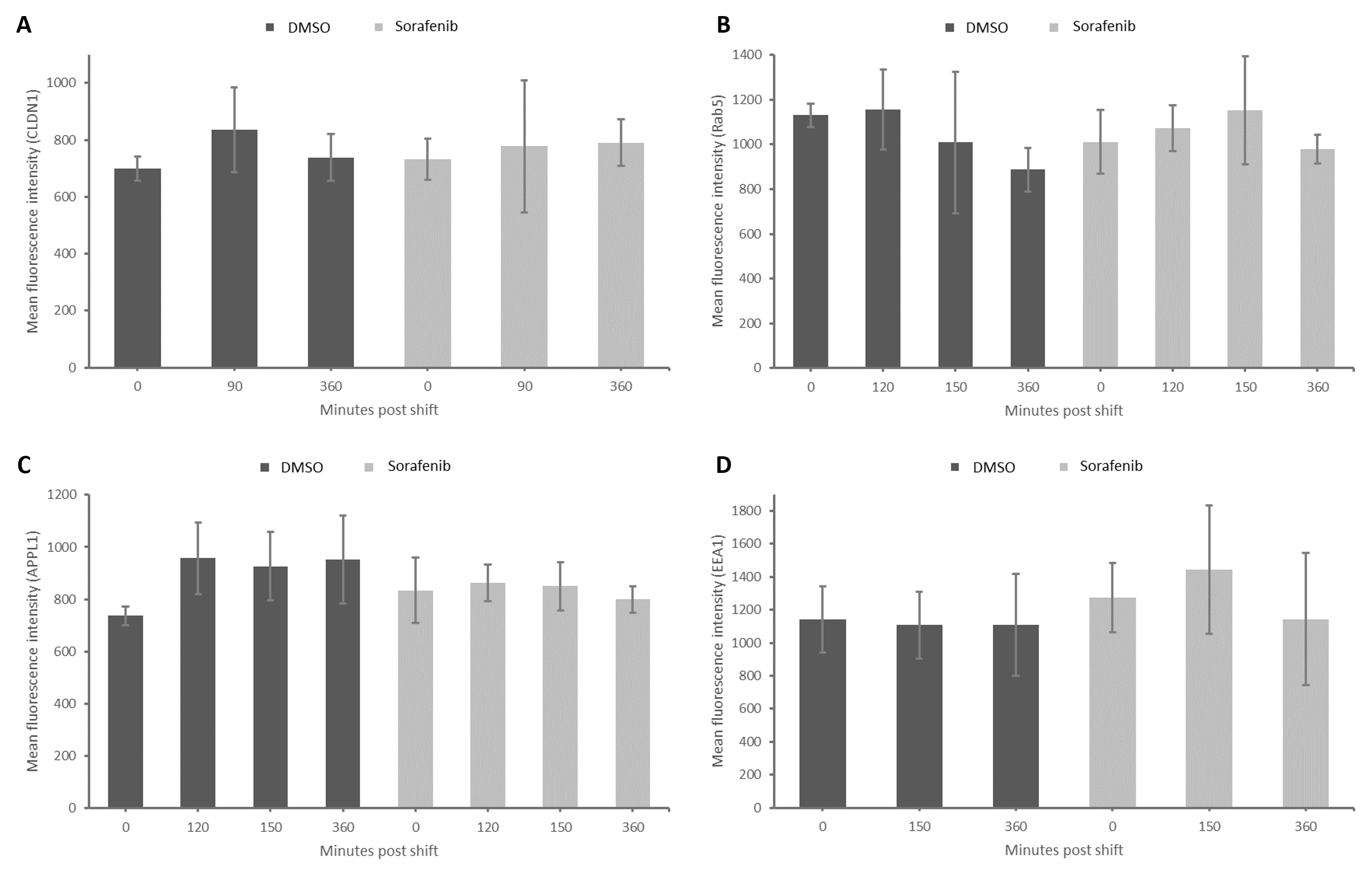

Supplement: S3 Fig — (A-D) Huh-7.5 spheroids were incubated with DMSO or 5 μM sorafenib for 2h, infected with DiD-HCV (red) with DMSO or sorafenib for 1 hr at 4°C, shifted to 37°C for the indicated times, fixed, and probed for CLDN1 (A), Rab5 (B), APPL1 (C) or EEA1 (D). Fluorescence intensity was measured. Mean +/- SD. (TIF) [file ppat.1011887.s003.tif]

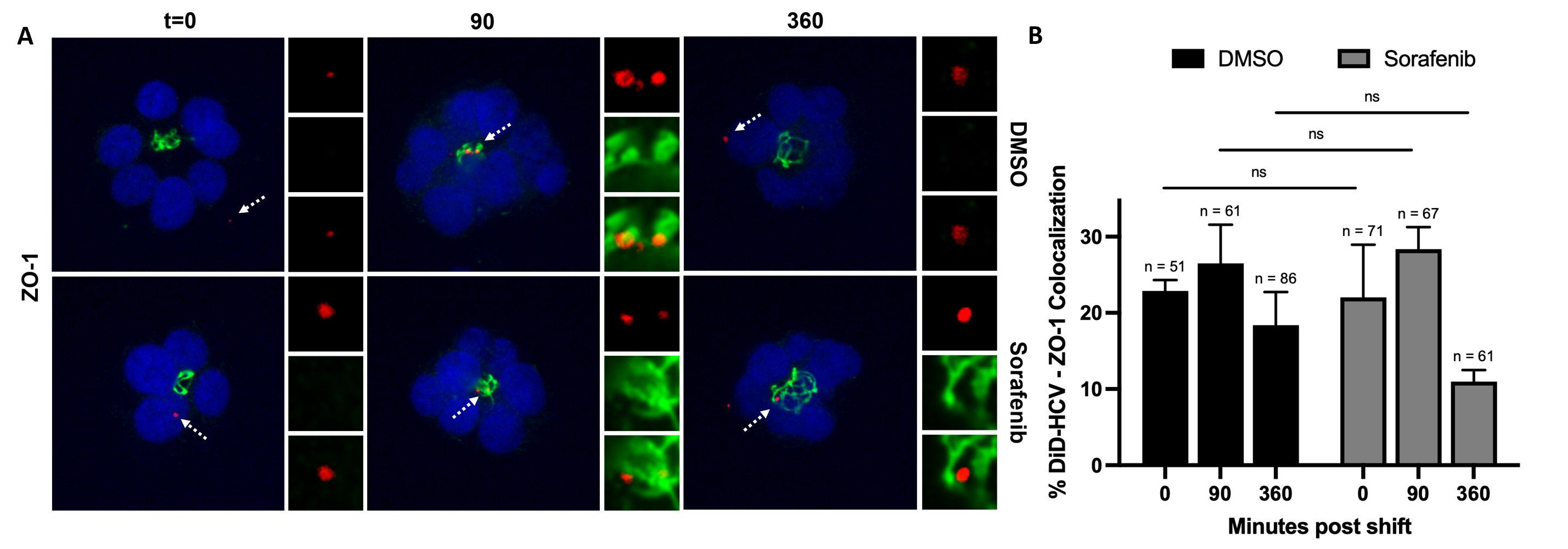

Supplement: S4 Fig — (A) Huh-7.5 spheroids were incubated with DMSO or 5 μM AG-1478 for 2h, infected with DiD-HCV (red) with DMSO or AG-1478 for 1 hr at 4°C, shifted to 37°C for the indicated times, fixed, and probed for ZO-1 (green). (B) Quantitation of (A). n = total DiD signal. Mean +/- SEM. (TIF) [file ppat.1011887.s004.tif]

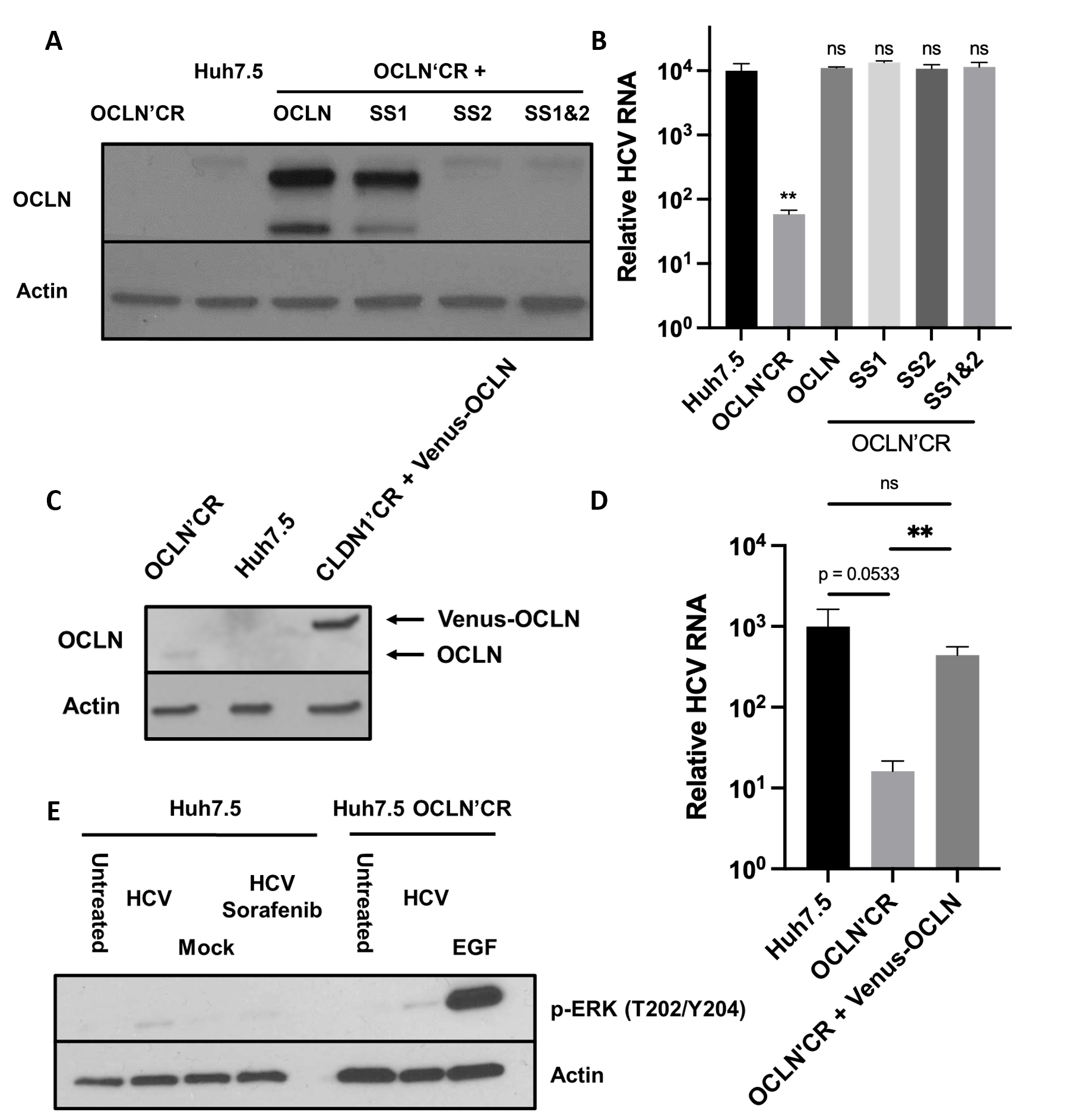

Supplement: S5 Fig — (A and C) Western blot of Huh-7.5 wildtype, OCLN CRISPR’ed, and complemented cells. (B and D) Huh-7.5 wildtype, OCLN CRISPR’ed, or complemented cells were seeded onto 96-well plates, infected with HCV for 48 hr, and then analyzed for relative HCV RNA levels. (E) Spheroids of Huh-7.5 wildtype or OCLN CRISPR’ed cells were serum starved, incubated with 5 μM sorafenib (if indicated) for 2 hr, infected with concentrated HCV with sorafenib (if indicated) for 1 hr at 4°C, shifted to 37°C, processed with Matrigel cell recovery solution, and lysed at 120 min post temperature shift. For EGF-treated sample, spheroids of Huh-7.5 wildtype cells were serum starved, stimulated with 40 ng/mL EGF, processed with Matrigel cell recovery solution, and lysed at 60 min post EGF stimulation. Lysate samples were immunoblotted for the indicated proteins. Mean +/- SD. **p < 0.01. (TIF) [file ppat.1011887.s005.tif]

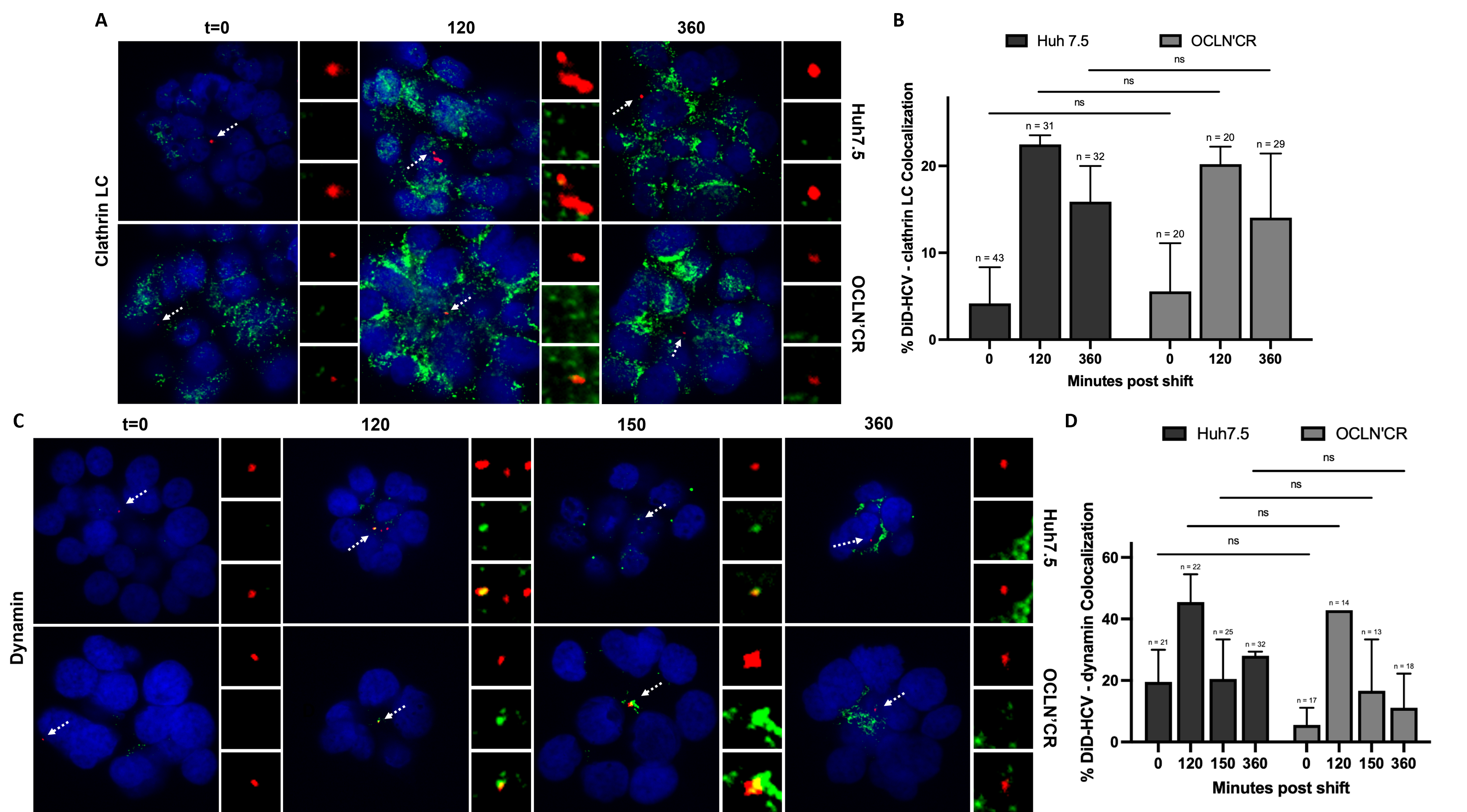

Supplement: S6 Fig — (A and C) Spheroids of Huh-7.5 wildtype or OCLN CRISPR’ed cells were infected with DiD-HCV (red) for 1 hr at 4°C, shifted to 37°C for the indicated times, fixed, and probed for clathrin light chain (clathrin LC) (A) or dynamin (C) (green). (B and D) Quantitation of (A) and (C), respectively. n = total DiD signal. Mean +/- SEM. (TIF) [file ppat.1011887.s006.tif]
